# Why Do Parents Bring Their Children to the Emergency Department? A Systematic Inventory of Motives

**DOI:** 10.1155/2015/978412

**Published:** 2015-11-04

**Authors:** Anne Costet Wong, Isabelle Claudet, Paul Sorum, Etienne Mullet

**Affiliations:** ^1^Pediatric Emergency Department, Hôpital des Enfants, 330 avenue de Grande-Bretagne, 31059 Toulouse Cedex 9, France; ^2^Internal Medicine & Pediatrics, Albany Medical College, 724 Watervliet-Shaker Road, Latham, NY 12110, USA; ^3^Department of Ethics of the Institute of Advanced Studies (EPHE), Maison de la Recherche, UTM, 5 allées Antonio-Machado, 31058 Toulouse Cedex 9, France

## Abstract

Parents frequently bring their children to general or pediatric emergency departments (EDs), even though many of these visits are judged by others to be “nonurgent” and inappropriate. This study examined the motives behind parents' decisions to take their children to a pediatric emergency department (PED). At a PED in Toulouse, France, 497 parents rated their level of agreement with each of 69 possible motives—representing all categories of human motivation—for coming to the PED that day. Exploratory and confirmatory factor analyses found evidence for six separable motives, called (in order of importance) (a) Seeking Quick Diagnosis, Treatment, and Reassurance; (b) PED as the Best Place to Go; (c) Empathic Concern for Child's Suffering; (d) Being Considered by Others as Responsible Parents; (e) External Factors; and (f) Dissatisfaction with Previous Consultation. *Conclusions*. Parents' motives in bringing their children to the PED are primarily serious and goal-oriented. They are also often emotion based, as would be expected in parents of ill children. The parents would be unlikely to agree that these visits were inappropriate.

## 1. Introduction

Emergency department (ED) use is common for both children and adults not only in a country without universal health insurance like the United States [1] but also in countries like France where virtually all residents have health insurance. In 2006 the annual rate of ED visits for Parisian children less than 2 years old ranged, according to district, from 46.9 to 91.3 per 100 children [2].

Many of these visits are not for true emergencies but for what ED physicians, insurers, policy makers, and researchers consider as “inappropriate” or “nonurgent,” that is, minor complaints that could have been dealt with in primary care offices on either that or the following day. Durand and colleagues [3] found that, in studies in a variety of countries around the world, the estimates of nonurgent visits by adults and children ranged from 4.8% to 90%, with a median of 32%. One reason for the variation was the inconsistency in defining nonurgent [3], as pointed out also by Mistry and colleagues [4] for pediatric ED visits. Recent figures on pediatric nonurgent visits include 58% in the United States [5], 57.1% in Italy [6], and 39.9% in Belgium [7]. In many cases, parents do not even try to contact a primary care physician [7–9]. Furthermore, they go to EDs even though they often have long waits; their children are likely to undergo more testing and treatment than by their own doctors; the ED physicians do not know their medical histories and usually do not provide follow-up; and in many cases the costs to them will be higher.

Many researchers have, therefore, tried to understand why parents decide to bring their children to EDs. They hope to find out how to reduce unnecessary ED visits and thereby how to improve continuity of care [10]; to lessen overcrowding in EDs, distraction from true emergencies, and diversion of ambulances to less crowded but more distant EDs [11, 12]; and to decrease health care expenses [6, 11, 13, 14].

Reasons given by parents for their decisions to go to pediatric EDs (PEDs), even without referral by their children's primary care physicians, include that they seek a quick and convenient solution to a health issue [1, 8, 10, 15–18]; that they view the PED as the best place to go because of its better physicians and/or medical equipment [1, 5, 7, 8, 10, 17, 19]; that they are dissatisfied with their primary care physician's office or the physician's diagnosis and/or treatment [8, 15, 20]; that they are very worried about their child's health [8, 15, 17, 21]; that they are in financial straits [17, 20]; that they have a habit of going to the ED [1, 7, 8]; and that they have no access to other care [1, 17, 19]. Adult patients have given similar reasons [13, 22].

The methodologies of the above-mentioned studies were not based on a broad theory of human motivation and may, as a result, have neglected important motives. In this study, therefore, we examined parental motives by applying Michael Apter's metamotivational theory (MTT) [23, 24], which encompasses and systematizes the great diversity of human motives. By including all theoretically possible categories of motives in the questionnaire, we could ensure that it was psychologically complete, that is, that no possible parental motive was missed. MTT is described in more detail in the Appendix.

Objectives of the study were (a) to inventory the motives for going to PEDs; (b) to order these motives, or categories of motives, as a function of their importance; and (c) to find out how demographic characteristics were associated with these motives.

## 2. Materials and Methods

The study was approved by the Ethics Committee of the Children's Hospital of Toulouse.

### 2.1. Participants

The participants were parents attending the PED of the Children's Hospital, a tertiary-level pediatric hospital in Toulouse, France. The research assistant invited the parents of every child to participate, informed them about the survey, and obtained oral consent from those who agreed to participate. The Ethics Committee required only oral consent.

### 2.2. Material

The first questionnaire contained 69 items referring to motives for going to a PED (Table 1). Forty-eight items were created by the members of the research team, inspired by MMT (see Table 5), by the medical literature (cited above), and by their personal experience as physicians. Items were purposely and systematically created to correspond to all of MMT's categories of motives. This questionnaire was then presented to a focus group of parents that reformulated items judged as ambiguous and suggested additional items based on their personal experiences as parents. The new questionnaire was presented to another focus group, and the process was repeated until saturation occurred. The parents in the focus groups were contacted at the PED. Twenty-one additional items were created in this way. The common wording of all items in the questionnaire—“One of the reasons I came to the PED today with my child”—was chosen to acknowledge that several motives could be operating at the same time.

The second questionnaire asked about demographics, about previous ED use, and about prior doctor visits during this illness.

### 2.3. Procedure

Participants responded individually in the waiting room, before their children were seen. If both parents were present, only one filled out the questionnaires but that one could confer with the other. The research assistant was, in most cases, not present during the process in order not to influence them. They indicated how much they agreed with each statement on the first questionnaire by putting a mark on the 10-point response scale under each one. The two extremes of the scales were labeled “complete disagreement” and “complete agreement.” They then completed the second questionnaire. The process took approximately 30 minutes.

The treating physician subsequently indicated the severity of each child's illness—on a scale of 1 (not severe), 2 (mildly severe), or 3 (severe)—and whether the child was hospitalized.

### 2.4. Analyses

The character of this study was both exploratory and confirmatory. Through exploratory factor analyses using two-thirds of the sample, a motivational structure was delineated. Through confirmatory factor analyses using the other third, the robustness of this motivational structure was assessed.

Exploratory factor analysis is a statistical technique that is used to reduce a number of variables into a smaller set of unobserved factors. The variables within each factor are empirically highly correlated with each other but not with the other variables. If the correlation between items and any factor was lower than 0.30 and their means were very low, the items were removed from the analysis. Confirmatory factor analysis is a statistical technique used to test whether a set of factors that has been obtained through exploratory factor analysis of data from one sample fits the data observed in another sample. As the exploratory factor analysis has to be conducted on all the useable items, the confirmatory analysis should only be done on a representative and more manageable subset of items, namely, on the three items with the highest “loadings” (the highest correlations with the factor) within each of the factors found in the exploratory analysis. Therefore, the exploratory factor analysis requires a larger sample size than the confirmatory analysis, calculated in this case to be 2 to 1 [25].

Linear correlations were computed between factor scores and demographic characteristics.

The sample size of 500 was determined in accordance with the technical requirements of factor analysis for a maximum expected number of factors of 10. Our choice of 10 was based on our analysis of the previous studies of reasons to go to the ED.

## 3. Results and Discussion

### 3.1. Results

The participation rate was 65%. Three questionnaires were not fully completed; 497 were included in the analyses. All families were insured under France's system of universal health insurance. Children's mean age was 6.6 years; 29% were less than 2. Demographic characteristics are shown in Table 2.

Table 1 shows the mean ratings (on a scale of 1 to 10) of each item in the exploratory factor analysis using the first subsample (*N* = 332). Thirteen of the 69 items did not load on any factor (correlation between item and any factor < .30), and their means were very low. Therefore, they were removed from the analysis. A second exploratory factor analysis was conducted on the 56 remaining items. Six interpretable and independent factors emerged, namely, (1)* Being Considered by Others as Responsible Parents* (which explained 13% of the variance); (2)* Empathic Concern for Child's Suffering* (8% of the variance); (3)* Seeking Quick Diagnosis, Treatment, and Reassurance* (12%); (4)* Dissatisfaction with Previous Consultation* (7%); (5)* PED as the Best Place* (7%); (6) and* External Factors* (5%), such as the family physician's absence, the impossibility of borrowing money, and a recommendation from the service that provides urgent home visits.

The results of the confirmatory factor analysis, conducted on the three items with the highest loadings on each of the six factors using the second subsample (*N* = 168), are shown in Table 3. The model derived from the first set closely fits the second set of data. The mean scores for each factor in the final model—on a 10-point scale—range from 1.79 (*Dissatisfaction with Previous Consultation*) to 6.82 (*Seeking Quick Diagnosis, Treatment, and Reassurance*).

Table 4 shows correlations between demographic characteristics, severity indices (as subsequently judged by physicians), and motives. Seeking quick diagnosis, treatment, and reassurance was significantly more strongly endorsed by parents of one child than by parents of more than one. Considering the PED as the best place to go was given a higher rating by younger fathers than by older ones. Empathic concern for the child's suffering was given a higher rating by parents with only one child. Being considered by others as responsible parents was a stronger motive for parents of boys than of girls, for younger mothers, for less educated parents, and for parents who were more likely to go to EDs without first consulting their regular physician, and a stronger motive in cases judged by the treating physician to be of lesser severity. Finally, external factors were more strongly endorsed as motives by parents of older children who had siblings and in cases in which symptoms had more recently appeared.

### 3.2. Discussion

The aim of our study was to make an inventory of the motives of parents in bringing their children to PEDs using a broad theoretical framework. We found that, as in other studies [8, 15, 17, 21], parents endorsed a wide variety of motives. The consistency of the motives expressed in these different studies demonstrates their psychological robustness. We were able to separate them into six independent factors. Finding factors meant the guarantee that parents' responses were meaningful, that is, that their responses were sufficiently coherent such that statistical analysis could reveal a clear structure to them. The emergence of a clear factorial structure also enabled measurement of the strength of each motive.

The most highly rated factor was* Seeking Quick Diagnosis, Treatment, and Reassurance*. Parents who strongly endorsed this set of motives (described as “telic” in MMT) seemed to be highly goal-oriented, going to a PED to obtain care they saw as important. This is consistent with findings in other countries [8, 15, 17, 26]. Fieldston and colleagues [15, p. 222] described guardians as “worried that symptoms represented serious illness and that it was important to seek medical attention.” Similarly, Durand and colleagues [22], in their study of French adult patients, characterized them as “rational consumers” (in contrast to the “abusive and irresponsible consumers” portrayed by the ED health professionals they interviewed). These parents are unlikely, therefore, to agree with the charge that large numbers of visits to PEDs are unwarranted.

The second most highly rated factor was* PED as the Best Place to Go* (which would be called a “conformist” motive by MTT). Their respect for the PED may have been stimulated by television shows and by reports in the media about the technology and specialization available in hospitals (as well as, in the United States, by advertising). They may not have appreciated that technology and specialized care are not always beneficial to patients. This set of motives was rated highly by younger fathers in particular.

*Empathic Concern for Child's Suffering* received a lower overall rating. Those parents who strongly endorsed these motives (labeled as “sympathy” in MTT) were no longer able to put up with their children's suffering. They tended to be parents of a single child and, therefore, likely to be less experienced in coping with sick children. It is striking, however, that distress at their child's current suffering was much less important than the goal-oriented aim of obtaining the most effective treatment.

The other three factors were rated quite low overall but were endorsed by some parents.* Being Considered by Others as Responsible Parents* was rated highly by parents who seemed to be concerned about their own image as parents (labeled as “autic” in MTT). They likely viewed going to PEDs as demonstrating responsible and loving parenthood. These parents tended to be younger (especially the mothers) and less educated than other parents and to go to EDs for themselves more frequently. Their child's condition was not likely to be judged as bad by the ED physician.

*External Factors* was rated low. Physicians and health services experts, especially in the US, often cite structural factors as important determinants of excessive ED utilization [6, 11, 13, 15, 27–30]. Those few parents who endorsed these motives strongly indicated thereby that going to PEDs was not a truly personal decision. They followed the instruction of the urgent care service or could not go to another medical facility owing to the absence of a physician or to lack of money. Their child's illness tended to be more acute; that is, the child was more likely than others to have experienced pain and/or fever for less than 12 hours.

The lowest average rating was for* Dissatisfaction with Previous Consultation* (a “negativist” motive in the terminology of MMT). Only a few parents were very unhappy with the care provided by their family physicians, in accordance with findings in other countries [8, 31].

Our study has, of course, its limitations. It was conducted in a single site in France, and the motives of the 35% of parents who declined to participate are unknown. The study's results must, therefore, be generalized with caution to other populations of children and parents and to other sites. Furthermore, even if the questionnaire is based on a broad theory of motivation, it has not been validated and should, therefore, be used prospectively again to confirm these results. Nonetheless, our findings can help to illuminate the current debate about the “overuse” of EDs.

Access to care is a key issue for parents, as well as for adult patients, and, therefore, is a focus of reform efforts. The 2011 National Health Interview Survey in the United States [27] found that, among adults who went to EDs and were not admitted to the hospital (68.9% of those visiting the ED), 78.9% had had at least one “access issue” for going to the ED (defined as indicating “not having another place to go,” “doctor's office or clinic being not open,” “ED being the closest ‘provider',” and/or “clinic being not open”). It seems possible to reduce ED visits, therefore, by improving access to other sources of care (as in the state of North Carolina [32]). Without this, as the Medicaid experience in the state of Oregon showed, expanding insurance will not reduce ED use; it will increase it [33]. Accordingly, many reformers have proposed encouraging pediatric practices to expand the availability of urgent visits [10, 30]; France has tried to set up primary care units at or near EDs [34]; and Italy is requiring its primary care offices to be open 24 hours a day 7 days a week [6]. Wang and colleagues [35] tested in the United States a pilot PED diversion program for children with Medicaid—using extended office hours, multiple access locations, and care coordination—that reduced ED visits by 8 visits per 1000 members per month (compared with the control group). Because their diversion program was so expensive, however, the overall cost of care was not reduced. Our study demonstrates the limits or barriers, from the parents' perspective, to the success of such efforts.

We found that parents generally bring their children to PEDs with serious, goal-oriented motives. Similarly, in the United States, the 2011 National Health Interview Survey [27] found that 65.0% of adults not admitted to the hospital cited at least one “acuity issue” (defined as indicating “only hospital could help,” “advised by health provider to go to ED,” and/or “problem too serious for doctor's office or clinic”); Kubicek and colleagues [5] found that 63% of parents bringing their children for reasons assessed as nonurgent perceived the visit as “very” or “extremely” urgent; and Kalidindi and colleagues [36] found that parents assessed 94% of their visits as urgent, even though 27% of these “urgent” visits were assessed as nonurgent by the physicians. Thus, even if access issues were greatly reduced in the US and other countries, the acuity issues would persist.

Because there is no consensus between the population and physicians about what is urgent and what is not, staff members in the EDs tend to define many visits as nonurgent, to label them as inappropriate, and to feel frustrated [17, 31]. In general EDs in the United States, the presenting complaints of adult patients judged subsequently as primary care treatable do not in fact differ from those judged as needing ED care [37]. Furthermore, as Cresson [28, p. 448] states, “To speak of ‘false urgency' in situations where there is urgency for the patient and not for the doctors is to act as if the latter were the only ones capable of defining urgency.” If overworked ED physicians, as well as researchers and policy makers, focus on ultimate diagnoses and wasted resources, they forget that the parents do not have the medical knowledge to make these diagnoses, do not know what the final diagnoses will be, and do not know how the illnesses are going to evolve. As Adams concludes, trying to “discern low-acuity conditions and putting up barriers to receiving care or denying payment after receiving care will work no better in future generations than in the past” [38, p. 1174].

One logical solution would be to educate parents better [5, 10, 17] and specifically those parents whose own characteristics—for example, in a US study, single parents, those who were themselves brought to EDs as children, and those with Medicaid [39]—make them more likely to use the ED. These efforts could involve targeted messaging to high utilizers [40] or more formal instruction, but so far success has been limited. In one study [41], 130 families were randomly assigned to receive, or not, an educational intervention in the PED that involved reviewing with a research assistant a booklet about how to manage minor illnesses and watching a 10-minute video. At 6 months there was no difference between groups in PED utilization, and most visits were still for minor illnesses. Similarly, a 90-minute, interactive educational activity at primary care offices increased 32 parents' knowledge of fever, colds, and minor trauma and increased their after-hours telephone use; but it did not decrease their ED use [42].

Education may, of course, have greater effects on other groups of parents than those studied and if done more intensively and repeatedly. Yet our findings point to one possible reason for the limited impact of greater knowledge: the importance for many parents of emotional motives—of anxiety about and empathetic concern for the child (as would be expected of parents) as well as, for some, of a desire to be seen as a caring parent. The setting able immediately to soothe their anxiety and distress is the hospital and its emergency department. It is not surprising, therefore, that the rate of ED visits has been increasing even in countries, like France, that have extended basic insurance to all residents.

## 4. Conclusion

When parents in France, as elsewhere, think their children are suffering and might be seriously ill, they are goal-oriented and, often, frightened. They bring their children to the places that are immediately available and that, they think, are most capable of helping their children, to the PEDs. These reactions are normal and hard to change. Thus, as Hendry, Beattie, and Heaney in the United Kingdom concluded, “perhaps it is the service design, rather than patient behavior, that is inappropriate” [8, p. 632]. The solution is not only to provide anxious parents with expanded daytime sick visits, 24-hour telephone access, and even night-time home visits, as is currently possible in many places [43]. The solution may also be to expand the PEDs, that is, to provide urgent visit capacity for minor illnesses and injuries, at lower cost than truly urgent visits, in the places of reassurance and security, the PEDs themselves.

## Figures and Tables

**Table 1:** The 69 items used in the questionnaire. Means and standard deviations of the responses. The items have been ranked from the most strongly endorsed to the least strongly endorsed.

One of the reasons I came to the PED today with my child is that...	M	SD
I wished my child's pain to be treated quickly	7.52	3.32
I was anxious for my child	7.38	3.22
I wanted quickly to have a diagnosis for my child	7.21	3.44
I wished my child no longer to suffer	6.71	3.55
I wished my child's dizzy spells to be treated quickly	6.65	3.74
I wished my child to benefit from all available care	6.60	3.44
I wished my child to benefit from the best treatments available	6.55	3.58
ED has all kinds of medical equipment in place	6.45	3.50
I wanted quickly to get treatment for my child	6.41	3.58
I feared that my child's illness was very severe	6.37	3.41
I was personally very anxious	6.23	3.53
My child's health seemed to deteriorate	5.96	3.67
I was not able to put up with seeing my child suffering	5.94	3.72
The caregivers in this department are the most specialized	5.88	3.51
I wished to know more about my child's illness	5.69	3.91
The caregivers in this department are the ones who are the most qualified	5.47	3.63
I wished to be personally reassured	5.42	3.62
My child was no longer able to put up with the suffering	4.76	3.67
My family physician told me to go to the ED	4.60	4.14
Something had to be done	4.44	3.69
I felt the situation had escaped my control	4.14	3.49
The usual treatment I gave to my child was not effective	3.95	3.67
The family physician was absent	3.88	3.79
I wished my child to have X-rays quickly	3.87	3.70
I wished my child to be reassured quickly	3.86	3.51
I was frightened by my child's elevated fever	3.77	3.55
I did not want to let things go on forever as some other parents do	3.46	3.35
I wanted my child's fever to be brought down	3.44	3.37
My child illness was going on forever	3.41	3.46
I wished my child's fever to diminish	3.21	3.30
I was no longer able to put up with seeing my child with an elevated fever	3.21	3.22
I wanted to show that I am responsive to my child's health status	2.97	3.31
The family physician did not respond when I called her	2.94	3.31
I wanted to have alternative advice about my child's health	2.82	3.07
I feel myself partly responsible for my children's bad health	2.79	3.10
Number 15 [the emergency service] told me to go to the ED	2.78	3.32
My partner was very concerned about our child's bad health	2.50	2.77
I wished my child to be aware that I care about his/her health	2.44	2.85
My child was no longer able to put up with such an elevated fever	2.37	2.71
The family physician did not respond to my call and I was afraid my child was in need of surgery	2.26	1.75
I have no regular physician and the SAMU told me to go quickly to the PED	2.23	2.19
The PED is the closest medical facility to my home	2.22	2.63
I wanted my child to return to school quickly	2.17	2.60
I wished that nobody could say I neglected my child	2.15	2.57
I wanted my child to have some laboratory exams quickly	2.09	2.39
I was told that the PED has a good reputation	2.06	2.45
I wished my child not to be deprived of the outdoor activities he/she likes	2.05	2.45
I thought my child needed an operation quickly	2.05	2.39
It is what I always do	1.94	2.31
I considered that my child had not been correctly treated by our family physician	1.93	2.23
I wanted to be viewed as a good parent	1.90	2.36
I wanted my child to realize he/she was seriously ill	1.86	2.30
I considered that the family physician's diagnosis was wrong	1.85	2.07
I wished my partner to be reassured quickly	1.74	2.03
I did not wish my child to be as severely ill as I had been in the past	1.74	2.05
I wished my child to have nothing to reproach me for in the future	1.71	2.06
I have no family physician	1.69	2.02
I wanted to prove to others that my child's illness was serious	1.68	1.94
I have seen on TV the way PEDs work	1.67	1.96
I considered that the family physician had erred in his prescriptions	1.61	1.68
I just wanted to locate the ED in case I need to go there in the future	1.45	1.65
There is currently an epidemic and I wished to know my child's health status	1.41	1.57
I wanted to prevent possible criticisms from the family	1.40	1.56
I did not have the money on hand for a regular office visit	1.36	1.45
It is what everybody does	1.30	1.33
My family physician told me she was wrong	1.21	0.92
I was very interested in seeing the way PED works	1.19	1.03

**Table 2:** Demographic characteristics of the sample.

Characteristic	Level	Number (percentage)
Gender	Girls	215 (43)
	Boys	285 (57)

Family	Live with their two parents	415 (83)
	Live with their mother	75 (15)
	Live with someone else	10 (2)

Parents' presence	Both parents are present	395 (79)
	One parent is present	105 (21)

Parents' mean ages	Mother	34.60
	Father	37.00

Health insurance	No supplementary insurance	15 (3)
	Supplementary insurance through employers	430 (86)
	Insurance through the state-financed program for poor people	55 (11)

Delay between symptom onset and PED visit	Less than 12 hours	265 (53)
	Between 12 and 24 hours	85 (17)
	More than 24 hours	150 (30)

Prior visit to a physician	Family physician	85 (37)
	Pediatrician	50 (10)
	Mobile doctor service^*∗*^	20 (4)
	No	250 (50)

Number of previous visits to the PED^*∗∗*^	None	165 (33)
	One	160 (32)
	Two	135 (27)
	More	40 (8)

Type of health problem	Illness	265 (53)
	Trauma	160 (32)
	Other	75 (15)

Decision to hospitalize	No	455 (91)
	Yes	45 (9)

^*∗*^The mobile doctor service will make urgent visits to patients' homes on nights and weekends in response to telephone calls from the patients.

^*∗∗*^As indicated by the parents.

**Table 3:** Results of the confirmatory factor analysis. The global fit index value was .88; the comparative fit index value was .93; the root mean square error of adjustment value was .07 [.05–.08]; the Chi² value was 218.54, *p* < .001; and the Chi²/df ratio was 1.82. Correlation coefficients of each item with each factor.

One of the reasons I came to the PED today with my child is that...	Factors						Error	*t*
	SQT	BP	ECS	CRP	EF	DPC		
I wished my child's pain to be treated quickly	.66						.06	11.29
I wished my child no longer to suffer	.81						.05	15.49
I was personally very anxious	.55						.07	8.33
Caregivers in this department are the ones who are the most qualified		.85					.03	32.45
Caregivers in this department are the most specialized		.84					.03	29.71
The PED has all kinds of medical equipment in place		.95					.02	50.17
I was frightened by my child's elevated fever			.79				.04	22.36
I wanted my child's fever to be brought down			.88				.03	30.70
I was no longer able to put up with seeing my child with an elevated fever			.87				.03	29.71
I wanted to be viewed as a good parent				.90			.04	25.20
I wished my child to have nothing to reproach me for in the future				.77			.04	18.02
I wanted my child to be aware that I care about his/her health				.74			.04	16.84
I do not have the money to put down for a regular office visit					.56		.07	8.19
I have no regular physician and the emergency medical service told me to go quickly to the PED					.70		.06	11.14
The family physician did not respond to my call, and I was afraid my child was in need of surgery					.80		.06	13.10
I considered that my child had not been correctly treated by our family physician						.58	.06	10.05
I considered that the family physician's diagnosis was wrong						.96	.04	21.56
I considered that the family physician had erred in his prescriptions						.75	.05	15.46

Mean rating of each factor	6.82	5.80	3.47	2.02	1.95	1.79		
Standard deviation	2.72	3.17	3.07	2.12	1.42	1.77		
Scores ≥ 6.0^*∗*^	69%	52%	23%	7%	3%	6%		
Internal validity (alpha)	.69	.82	.90	.85	.68	.87		

SQD = Seeking Quick Diagnosis, Treatment, and Reassurance, BP = PED as the Best Place, ECS = Empathic Concern for Child's Suffering, CRP = Being Considered by Others as Responsible Parents, EF = External Factors, and DPC = Dissatisfaction with Previous Consultation.

*t* is a measure of significance; values > 1.96 are considered significant.

^*∗*^To indicate how many ratings in each factor were quite high (≥6 on a scale of 1–10).

**Table 4:** Correlations between motives, demographic characteristics, and severity indices.

Characteristics	Motives					
	SQD	BP	ECS	CRP	EF	DPC
Gender	−.09	−.08	−.02	−.24^*∗*^	.09	.05
Child's age	−.06	−.02	.03	−.09	.24^*∗*^	.05
Mother's age	−.14	−.13	−.11	−.22^*∗*^	.12	.04
Father's age	−.09	−.22^*∗*^	−.08	−.07	.11	.03
Siblings	−.20^*∗*^	−.05	−.21^*∗*^	.05	.26^*∗*^	.14
Level of education	−17	−.05	−.09	−.19^*∗*^	−.10	−.04
Number of previous consultations	.01	.05	−.03	.12	−.03	−.00
Number of parents' ER visits in total	−.03	.04	−.03	.21^*∗*^	.09	.01
Complete documentation	.15	.13	.08	−.00	−.17	−.02
Time since first symptoms	.13	.08	.18	−.04	−.31^*∗*^	.04
Level of severity	−.06	−.05	−.01	−.21^*∗*^	.10	.18
Subsequent hospitalization	.09	.06	.14	−.15	−.17	−.18

SQD = Seeking Quick Diagnosis, Treatment, and Reassurance, BP = PED as the Best Place, ECS = Empathic Concern for Child's Suffering, CRP = Being Considered by Others as Responsible Parents, EF = External Factors, and DPC = Dissatisfaction with Previous Consultation.

For ratings and ages (both continuous variables), classical Pearson correlations were computed; for ratings and the other, dichotomous variables, biserial correlations were computed.

^*∗*^ *p* < .05.

**Table 5:** Domains and states in metamotivational theory. Examples of corresponding possible motives for going to a pediatric emergency department (PED).

Domain	State	Characteristic	Possible motive for going to PED
Goals and means	Telic	Focusing on goals and achievement, with a serious attitude	Seeking quick solution to the problem (quick diagnosis and treatment)
	Paratelic	Focusing on the situation or the activity itself and on present moment, with a playful attitude	Having seen how PEDs work on the TV and feeling curiosity about it

Rules and constraints	Conformist	Following social codes, rules, and laws; showing respect or obedience; and adopting a conventional attitude	Believing that PED is the best place to go: PED has the best physicians and the best equipment
	Negativist	Opposing social expectations and rules; expressing hostility or dissidence; and adopting an unconventional attitude	Considering that the family physician's diagnosis was wrong

Transactions or exchanges with other people, things, and situations	Mastery	Trying to dominate people, things, or situations	Realizing that the situation was escaping one's control
	Sympathy	Feeling affection toward other people or things	Feeling strong compassion for one's suffering child

Relationships with other people, things, and situations	Autocentric	Being the focus of others' concerns and interests	Wanting to be considered as a good parent by child and others
	Intra-autic	Focusing on one's own concerns and interests	Feeling personally very anxious and worried
	Allocentric	Identifying with and focusing on the needs and interests of others	Wishing that child's high fever and suffering would be reduced

## Appendix

### Apter's Metamotivational Theory (MTT)

MMT classifies people's ways of dealing with the world according to four fundamental domains: goals and means; rules and constraints; transactions or exchanges with other people, things, or situations; and relationships with other people, things, or situations. As shown in Table 5, each domain is characterized by two or more possible states of mind, each of which corresponds to one category of motives.

## Abbreviations

ED: Emergency department
MMT: Metamotivational theory
PED: Pediatric emergency room.
